# Response from Savioli and Colleagues from the Department of Neglected Tropical Diseases, World Health Organization

**DOI:** 10.1371/journal.pmed.0030283

**Published:** 2006-06-27

**Authors:** Lorenzo Savioli, Dirk Engels, Denis Daumerie, Jean Jannin, Jorge Alvar, Kingsley Asiedu, Marc Gastellu-Etchegorry, Pere Simarro, Silvio P Mariotti

**Affiliations:** **1**World Health OrganizationDepartment of Control of Neglected Tropical Diseases, GenevaSwitzerland; **2**World Health OrganizationChronic Disease Prevention and Management, GenevaSwitzerland

We have read the article by Hotez et al. [ 1] and the letter by Torreele et al. [ 2].

The priority today is immediate action to expand delivery of effective tools and to strengthen the capacity of health and innovative delivery systems in the poorest sections of endemic countries to tackle the control, elimination, and eradication of neglected tropical diseases (NTDs). To this end, it is extremely important that all these neglected diseases be placed on the global public health agenda.

Approximately 1 billion people—one person in every six—suffer from one or more NTDs, such as Buruli ulcer, cholera, cysticercosis, dengue and dengue hemorrhagic fever, dracunculiasis (Guinea-worm disease), food-borne trematode infections, hydatidosis, leishmaniasis, lymphatic filariasis, onchocerciasis, schistosomiasis, soil-transmitted helminthiasis, trachoma, Chagas disease, and human African trypanosomiasis. Several of these diseases are vector borne. Some diseases affect individuals throughout their lives, causing a high degree of morbidity and physical disability and, in certain cases, gross disfigurement. Others are acute infections, with transient, severe, and sometimes fatal outcomes. Patients can face social stigmatization and abuse, which only adds to the already heavy disease burden. The common denominator of all the NTDs is that these diseases are invariably the diseases of the poorest in low-income countries.

For the majority of these diseases, inexpensive or donated drugs are available for their prevention and control or are part of strategies for control and elimination. These, when used on a large scale, are able to wipe out the burden caused by these ancient scourges of humanity. For leprosy, treatment with effective antibiotics, now kindly donated by Novartis, is leading to the elimination of this ancient disabling disease. In the case of blinding trachoma, the use of the recommended SAFE strategy (surgery, antibiotic therapy, facial cleanliness, and environmental improvement), including an effective antibiotic, donated by Pfizer through an ad hoc initiative (the International Trachoma Initiative), is enhancing the progress towards final elimination. Large-scale, regular treatment plays a central role in the control of many NTDs such as filariasis, onchocerciasis, schistosomiasis, and soil-transmitted nematode infections. For example, regular chemotherapy against intestinal worms reduces mortality and morbidity in preschool children, improves the nutritional status and academic performance of schoolchildren, and improves the health and well-being of pregnant women and their infants.

There is a second group of NTDs for which the only clinical option currently available is systematic case-finding and management at an early stage. These diseases include Buruli ulcer, Chagas disease, cholera and other diarrhoeal diseases, human African trypanosomiasis, and leishmaniasis. Simple diagnostic tools and safe and effective treatment regimens urgently need to be developed for these diseases. However, even for these infections, systematic and widespread use of the present “imperfect” tools at an early stage of disease can dramatically reduce mortality, morbidity, and disability. For others, vector-control tools are available and present the main method for successful transmission control, as in the case of Chagas disease.

There are examples of great successes in the fight against NTDs in both these groups, and these offer optimism for the future. Since 1985, 14.5 million patients have been cured of leprosy through multidrug therapy; today, fewer than 1 million people are newly affected by the disease. Before the start of the Guinea-worm Eradication Programme in the early 1980s, an estimated 3.5 million people were infected with the disease in 20 endemic countries. In 2005, only about 10,000 cases were reported in nine endemic countries, and the programme is moving towards eradication by 2009.

The control of onchocerciasis has freed more than 25 million hectares of previously onchocerciasis-infested land and made it available for resettlement and agricultural cultivation, thereby considerably improving rural development prospects in Africa and Latin America. During the last years, thanks to public–private partnerships with sanofi-aventis, human African trypanosomiasis control activities have increased, raising the total number of people screened through active case-finding and subsequently increasing the access to diagnosis and treatment of affected populations. These constant efforts have led to a substantial and regular decline in the number of new cases. The number of people infected, which were estimated at 300,000 cases in 1995, has been reduced to 50,000–70,000 in 2005 [ 3].

In other words, the area of NTDs is not only an area lacking drugs and tools that can effectively treat affected individuals and communities, but an area of action. As an example, praziquantel, a very effective, safe, and relatively cheap single-dose drug (approximately 20 Euro cents per dose) to treat schistosomiasis, affecting in Africa alone at least 160 million people, is not accessible to those in need due to lack of financial resources to purchase and deliver it. We also have a series of other effective antischistosomal drugs, such as oxamniquine and metrifonate, that could again be made available in case resistance to praziquantel were to develop. Triclabendazole, the only effective drug against fascioliasis, has been on the market for veterinary use for over 20 years and is still not widely available for human use. Other drugs to tackle onchocerciasis and lymphatic filariasis are generously given free by the producers, Merck and GlaxoSmithKline, but more funds are required to deliver them to the millions in need.

These and many other highly effective drugs developed in the late 1970s are now out of patent but still not available to poor communities. We are well aware that “market mechanisms” will never solve the problem of access to effective drugs in the poorest communities of the low-income countries. Therefore, drug donations and funds for drug delivery are needed to tackle a problem that is intimately linked to underdevelopment and marginalization.

Global health development policies must also be more balanced in allocating resources to research and control. For instance, the recent resolution of the European Parliament [ 4] is indeed a sign of great progress. However, this document tackles disproportionately the lack of tools and the need for research in drug development. We believe that—above all—priority should be given to generating resources to deliver the drugs already available to those in need while monitoring their use and efficacy.

WHO is expanding activities in this area. WHO has very recently developed guidelines towards effective integrated implementation of large-scale preventive chemotherapy strategies in consultation with Member States, academic organizations, and other partners. We believe these guidelines will be essential for Member States and interested non-governmental organizations to tackle the problem of NTDs in their countries on a large scale.

We agree that this need for immediate action in the medium- and long-term must be backed up by research and development of new drugs, vaccines (like those presently developed against hookworms), diagnostics, and other tools. We believe that focusing mainly on research and development at this stage is overshadowing the importance of reducing mortality, morbidity, and disability now with the existing technology.
